# Systemic inflammation and Oxidative stress with stroke mortality among patients admitted in tertiary Hospital in Uganda: a prospective cohort study in southwestern Uganda

**DOI:** 10.21203/rs.3.rs-3764472/v1

**Published:** 2024-01-08

**Authors:** Nicholas Kulaba, Adrian Kayanja, Josephine Naigaga, Jackson Lodiong Dumo, Josephine Najjuma, Mark Kaddu Mukasa, Elly T Katabira, Shirley M. Moore, Martha Sajatovic, Anthony Muyingo

**Affiliations:** Mbarara University of Science and Technology; Mbarara University of Science and Technology; Mbarara University of Science and Technology; Mbarara University of Science and Technology; Mbarara University of Science and Technology; Makerere University; Makerere University; University Hospitals Cleveland Medical center & Case Western Reverse University School of Medicine; University Hospitals Cleveland Medical center & Case Western Reverse University School of Medicine; Mbarara University of Science and Technology

**Keywords:** Inflammation, Oxidative stress, Stroke, Mortality

## Abstract

**Background:**

Stroke is an inflammatory state that causes death and chronic disability. Inflammation and oxidative stress are a predictor of poor clinical outcome, its effects are controversial and has not been evaluated in Sub-Saharan Africa (SSA).

**Methods:**

We conducted a prospective cohort study of CT head confirmed ischemic and hemorrhagic stroke admitted within 7 days of onset of motor weakness. Baseline CRP, NLR and baseline glucose was measured with subsequent modified Rankin Scale (mRS) score on day 14 post-stroke. Cox proportional hazard model was fitted to determine hazard ratios of mortality with CRP, NLR and blood glucose.

**Results:**

Out of 120 patients, 51.7% were female, 52.5% had ischemic stroke and the overall median age was 65 (IQR 54–80) years. Nineteen (15.8%) patients died within a median survival time of 7 days, while 32 (25.8%) died by day 14 after stroke.

**Conclusion:**

High C-reactive protein and stroke related hyperglycemia conferred statistically significant hazards of mortality among patients with acute and subacute stroke.

## Introduction

Stroke affects millions of people annually with a high mortality and morbidity (1, 2). There is a rise in stroke occurrence in low-income and middle-income countries (LMICs) (3). Hospital-based studies from Africa have shown a case fatality ranging from 16.2–46% among patients with stroke at 30 days (3). In Uganda, studies have shown a high 30 day mortality between 26.8% – 38.1% (4–6). Stroke has been described as a state of inflammation and oxidative stress (7).

Primary brain injury occurring in ischemia or hemorrhage is followed by secondary brain injury and Oxidative stress, which begins within minutes and persists for days to weeks or even longer (8). In primary brain injury; ischemic stroke refers to an abrupt cessation of blood supply in a vascular territory resulting in an ischemic core, surrounded by a penumbra and intracerebral hemorrhage refers to rupture of a blood vessel leading to extravasation of blood components directly into the brain parenchyma, forming a hematoma that provokes structural damage (8). Neural cellular damage and release of damage-associated molecular patterns (DAMPs) defines a common pathway that provokes an innate immune response characterized by neutrophils, microglia cell activity and adaptive immune response characterized by glial activation, recruitment of peripheral immune cells, and release of cytokines and chemokines (8, 9). The peripheral leucocytes infiltrate the injured brain aggravating further disruption of the blood brain barrier by releasing proinflammatory cytokines and reactive oxygen species (10, 11). An increased peripheral neutrophil count is independently predictive of severe stroke whereas lymphocytosis increases the regulation of anti-inflammatory cytokines like interleukin (IL) 10 and suppresses proinflammatory cytokines like IL 6 and tumor necrosis factor (TNF) alpha, thereby protecting the nerves (7, 12). Systemic pressure after stroke leads to low lymphocyte count through activation of the renin-angiotensin system, resulting in the release of cortisol and induction of lymphocyte apoptosis (13). A high NLR depicts neutrophilic elevation and lymphocytic depletion indicating a disproportionate interaction between central and peripheral inflammation. A high NLR is a negative prognostic indicator in acute ischemic stroke (AIS) and spontaneous intra-cerebral hemorrhage (ICH) (14). This differential cellular count is affected by the inflammatory process which can be assessed by inflammatory markers like C-reactive protein (CRP) (7). C-reactive protein (CRP) is a known biomarker of systemic inflammation among patients with stroke (15). A high CRP level is a predictor of mortality independent of stroke severity and infections (16). The level of brain inflammation is affected by the oxidative stress.

Oxidative stress defined as an imbalance between anti- and pro-oxidants, which has been implicated in the stroke pathogenesis (17). The brain is sensitive to oxidative damage because of its high and specific metabolic activity. High oxygen consumption, no energy reserves, almost exclusive oxidative phosphorylation, high lipid concentrations prone to peroxidation, and high levels of iron, all acting as a promotors of oxidative stress (18). Low blood flow reduces the amount of oxygen and glucose, following a cascade of events that leads to production of reactive oxygen species (ROSs) and free oxygen radicals that further worsen brain injury (17). Stroke related hyperglycemia mostly driven by stress hormones like cortisol, glucagon and adrenaline but some patients have underlying diabetes (19). Hyperglycemia subsequently induces intracellular ROS production resulting in an increased production of superoxide (20). Hyperglycemia exerts direct membrane lipid peroxidation and cell lysis in metabolically challenged tissue leading to global brain inflammation (19).

High biomarkers of inflammation and hyperglycemia are associated with poor short-term clinical outcomes (21). Studies on the association of inflammation and oxidative stress with all-cause mortality risk in patients with stroke have yielded inconsistent results. We set out to determine the effect inflammation and oxidative stroke on 14 day mortality among patients with stroke in Uganda

## Methods

### Study design and participants

This was a prospective cohort study of patients with acute and subacute stroke admitted to Mbarara Regional Referral Hospital, a tertiary hospital in South Western Uganda. We included patients; 18 years and above with a sudden onset of one sided neurologic deficits within 7 days and non-contrasted CT head confirmation of ischemic stroke evidenced by a hypodense lesion or hemorrhagic stroke evidenced by a hyperdense lesion in the brain. We excluded patients with traumatic intracerebral hemorrhage such as hematomas, and traumatic brain injury. At admission, all patients were positioned with head of the bed elevated at 30 degrees to prevent aspiration and oxygen saturation was kept above 93% as the standard of care. Blood pressure at admission was measured using EDAN M3^®^ (Edan USA 2014). Three blood pressure values were taken and an average of the last two was considered as the blood pressure at hospital admission (22). Socio-demographics (such as age, sex, marital status, address); behavioral factors (such as smoking and alcohol history) were captured. Past medical records were evaluated to capture history and duration of hypertension, diabetes mellitus, types of medications given, presence of co-morbid kidney disease and heart disease. A complete clinical examination was conducted which included; the Mayo Clinic Full Outline of Unresponsiveness score (FOUR score) to assess the level of consciousness and the National Institutes of Health Stroke Scale (NIHSS) score to assess stroke severity. Grading of the NIHSS was as follows; 0 = no stroke, 1–4 = minor stroke, 5–15 = moderate stroke, 16 to 20 = moderate to severe stroke, and 21–42 = severe stroke (23).

### Laboratory procedures at admission

Capillary blood glucose was measured using Accuchek glucometer (Roche Diagnostics Inc.). Full blood count was measured using Mindray hematology analyzer, and total Cholesterol (TC) was measured by Enzymatic linked immunosorbent assay method, using Human 200 analyzer (German Design, Human Diagnostics), renal function tests, serum Sodium and potassium were measured using Sysmex XNL-550^®^.

### Outcome Measures

The primary outcome of this study was defined as mortality at day 14 of stroke onset. Modified Rankin Scales (mRS), which is a measure of the degree of neurological disability or dependence on daily activities was in addition assessed as an outcome at day 14 of stroke among those who were still alive (24).

### Statistical Analysis

Clinical characteristics were computed as mean, and standard deviation for normally distributed variables. Categorical variables were summarized in frequencies and percentages. To determine the differences in baseline clinical characteristics between hemorrhagic stroke and ischemic stroke, were evaluated using a student’s t-test for continuous variables and chi-square test for categorical variables.

Cox-proportional hazard regression analysis was fitted to determine the hazard ratios of mortality at 14 days with 5% level of significance, 95% confidence interval and p - values. These were adjusted for baseline sociodemographic characteristics, Hypertension, type of stroke and NIHSS (stroke severity) against the clinical outcome (mortality at day 14)

## Results

**Figure 1:**
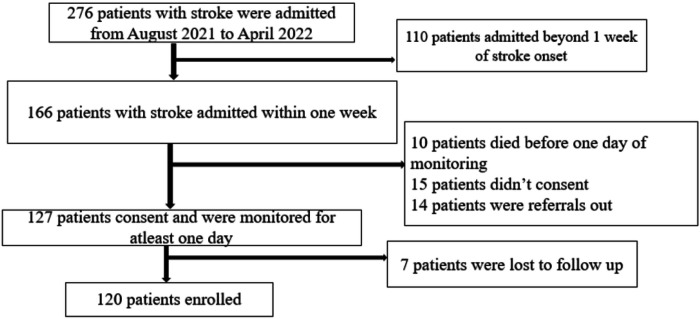
Study flow chart

Screened 276 participants with one sided neurological deficit between August 2021 and April 2022. We enrolled 120 patients with Computerized Tomography confirmed stroke (Fig. 1), 52.5% had ischemic stroke and 47.5% had hemorrhagic stroke (Table 1).

Out of 120 participants, female sex contributed 51.7% of the participants and the overall median age was 65 years (IQR: 54–80), 10.8% had diabetes mellitus, 43.3% had hypertension with 21.7% using anti-hypertensive medication and 7.5% were co-infected with HIV (Table 1). A history of smoking and harmful use of alcohol was elicited in 23.3% and 40.8% of all participants respectively (Table 1).

### Primary outcome:

The overall mortality derived from both hemorrhagic and ischemic stroke was 26.7% (32/120). The 14 day mortality was higher in hemorrhagic stroke (33.3%) than in Ischemic stroke (20.6%).

Using cox proportional hazard regression model in multivariate analysis, we included factors with p-value of less than 0.5 and those known parameters of inflammation. Patients with hyperglycemia of ≥ 10 mmol/L and high C – Reactive Protein ≥ 10 mg/L had high adjusted hazard ratios (aHR) of 3.8 (95% CI: 1.5–10.2), p = 0.007 and aHR = 9.2 (1.3–66.4), p = 0.028 respectively (Table 2). However NLR at admission had aHR = 1.1 (95%CI: 0.5–2.6), p = 0.816.

## DISCUSSION

In this study we set out to determine the effect of inflammation and oxidative stress assessed using C – Reactive Protein, random blood sugar and Neutrophil-lymphocyte ratio at admission among patients with acute and subacute stroke on mortality at day 14 of stroke onset. We found that stroke related hyperglycemia and high C – reactive Protein did significantly relate to mortality. Patients with hemorrhagic stroke had higher Neutrophil Lymphocyte Ratio (NLR) and C – Reactive Protein (CRP) in comparison with ischemic stroke but a lower random blood sugar (RBS) at admission. These findings support earlier studies that demonstrated that systemic inflammation and oxidative stress increase the risk of death and/or dependency after acute stroke. This study is among the few studies in sub-Saharan Africa that have evaluated inflammation and oxidative stress in stroke correlating it with mortality at day 14 of stroke onset.

Systemic inflammation described by a high Neutrophil Lymphocyte Ratio (NLR) which was not statistically significant but a high C- reactive protein (CRP) with significant adjusted hazards for mortality of 9.2 in our study. This implies that stroke is an inflammatory state and stroke related infections may also exacerbate the CRP value. Inflammation starts early and plays a central role not only in ischemic damage but also in endothelial progenitor cells in angiogenesis (2). When local inflammation occurs, it can worsen a secondary injury and evoke global brain inflammation (2). This means that higher baseline inflammation level is a good predictor of short-term poor outcome in strokes (25). C-reactive protein is a peripheral biomarker of inflammation and it is an acute phase protein which has been assessed as a biomarker for mortality and poor clinical outcome among patients with stroke (26). Yu et al found that a high CRP had a 2.07 fold risk of all-cause mortality in acute ischemic stroke (16). This study only assessed ischemic stroke patients yet in our study, patients with hemorrhagic stroke contributed a higher CRP than patients with ischemia. The odds for neurological improvement measured by mRS decrease as the level of plasma CRP increase after adjustment for age, sex, baseline neurological severity, and stroke subtypes (26). These findings may be partly explained by in increased risk of recurrent stroke and cardiac ischemia during the early period post stroke (27–29). Elevated CRP has been related to poor functional outcome, mortality and also to the occurrence of post-stroke infections (30). However even when patients with early infection are excluded during hospitalization, this does not significantly eliminate the association of CRP with mortality (31).

Stroke related hyperglycemia resulted in 3.2 high hazards of mortality among patients with stroke in our cohort yet only 11% had diabetes mellitus. The cause of the hyperglycemia in patients with stroke is mostly driven by stress hormones like cortisol, and catecholamines which play a big role in glucose regulation (32). Stroke induced hyperglycemia regardless of diabetes mellitus points to a physiological stress and also relative insulin resistance, which is linked to increased lipolysis (33). A study done in Egypt that was evaluating 24 hour hyperglycemia in stroke defined by blood sugar of 8.3 mmol/dl and found 1.2 adjusted risk for mortality among patients with stroke (34). This study had a lower cutoff compared to our study. These varying cutoffs for hyperglycemia in different studies among patients with stroke still show that stroke related hyperglycemia is strong predictor mortality (34). Experts recommend a target blood glucose between 7.8 mmol/L and 10 mmol/dl patients that have suffered a stroke (35). Tight glucose control (< 6.1mmol/dl) versus loose glucose control, there was no difference in the clinical outcome of patients with stroke (36). Persistent stroke induced hyperglycemia has been shown to decrease cerebral blood flow through vascular dilation leading to an increase intracranial pressure, causing cerebral edema, inflammation, and neuronal death hence resulting in blood brain barrier disruption, hemorrhagic transformation in acute ischemic stroke and growth of hematoma size in hemorrhagic stroke (34, 37–39).

In conclusion, we have provided evidence that high CRP and stroke related hyperglycemia in acute and subacute stroke were predictors of mortality. Setting up of stroke units in sub-Saharan Africa.

We recommend interventions for controlling systemic inflammation and blood sugars among patients with stroke to reduce mortality.

This study also has some limitations. Due to late presentation of patients to the hospital might have affected rate of inflammation and oxidative stress considering that the risk of infections from catheters and aspiration pneumonias are high during the late presentation hence producing a higher state of inflammation and oxidative stress.

## Figures and Tables

**Table 1:** Baseline clinical characteristics of patients with stroke.

Variable	Overall (N = 120)
Sex Female n (%)	62 (51.7%)
Age (Mean ± SD)	66 ± 16
Ischemic stroke n (%)	63 (52.5%)
Time to presentation (Median ± IQR)	4 (3–5)
Diabetes Mellitus n (%)	13 (11%)
Hypertension n (%)	52 (43%)
Smoking n (%)	28 (23%)
HIV n (%)	9 (8%)
Alcohol n (%)	49 (41 %)
NIHSS (Mean ± SD)	18 ± 9
SBP (mmHg) (mean ± SD)	153 ± 28
DBP (mmHg) (mean ± SD)	90 ± 18
RBS (mmol/L) (mean ± SD)	7.9 ± 3
CRP (mg/L) (mean ± SD)	67 ± 65
Neutrophil (mean ± SD)	6.8 ± 4
T-chol (mg/dl) (mean ± SD)	164.7 ± 82.7
Crea (mg/dl) (Mean ± SD)	1.5 ± 1.1
NLR (Mean ± SD)	5.3 ± 5.4

Crea - Creatinine, CRP- C-Reactive Protein, DBP- Diastolic Blood Pressure, HIV- Human Immunodeficiency Virus, NIHSS-National Institute of Health Stroke Scale, NLR- Neutrophil-Lymphocyte Ratio, RBS- Random Blood Sugar, SBP- Systolic Blood Pressure, SD- Standard

**Table 2:** Predictors of 14 day mortality among patients ivith stroke

Variable	Un adjusted			Adjusted		
	UHR	95%C.I	P-value	AHR	95%CI	P-value
**Fernale sex**	0.9	0.5 – 1.8	0.865			
**Age (≥65 years)**	0.8	0.4 – 1.6	0.482	0.6	0.3 – 1.5	0.287
**History of hypertension**	1.1	0.6 – 2.1	0.756			
**NIHSS ≥ 16 (Stroke severity)**	1.6	0.8 – 3.0	0.169	1.4	0.7 – 2.9	0.347
**Hemorrhagic stroke**	1.3	0.7 – 2.4	0.450	1.4	0.6 – 2.9	0.413
**NLR (≥3.5)**	1.2	0.6 – 2.3	0.560	1.1	0.5 – 2.6	0.816
**RBS at admission (≥10mmol/L)**	1.3	0.5 – 3.2	0.577	3.2	**1.5 – 10.2**	**0.007**
**CRP (≥10mg/L)**	2.3	1.3 – 3.9	0.004	9.2	**1.3 – 66.4**	**0.028**

CRP- C - reactive protein, NIHSS-National Institute of Health Stroke Scale, SDSBP-Standard deviation Systolic Blood Pressure NLR-Neutrophil Lymphocyte Ratio.

## Data Availability

Data available on request from the corresponding authors.

## Abbreviations

CRP: C - reactive protein
CT: Computed tomography
CVA: Cerebrovascular accident
MRRH: Mbarara Regional Referral Hospital
mRS: modified Rankin scale
NIHSS: National Institutes of Health Stroke Scale
NLR: Neutrophil Lymphocyte Ratio
